# Adherence with blood pressure self-monitoring in women with pregnancy hypertension, and comparisons to clinic readings: A secondary analysis of OPTIMUM-BP

**DOI:** 10.1016/j.preghy.2021.05.016

**Published:** 2021-08

**Authors:** Liza Bowen, Louise Pealing, Katherine Tucker, Richard J. McManus, Lucy C. Chappell

**Affiliations:** a Department of Population Health Sciences, King's College London, London, UK; b Department of Primary Care, Health Sciences, University of Oxford, UK; c Women and Children's Health, School of Life Course Sciences, King's College London, London, UK

**Keywords:** Blood pressure, Hypertension, Self-monitoring, Pregnancy, Adherence, SMBP, self-monitoring of blood pressure, CH, chronic hypertension, GH, gestational hypertension

## Abstract

**Objectives:**

To assess adherence to self-monitoring of blood pressure (SMBP), and differences between SMBP and clinic readings, in a self-monitoring intervention for managing pregnancy hypertension.

**Study design:**

OPTIMUM-BP was an unmasked randomised controlled clinical trial. 154 women with pregnancy hypertension from four maternity units in England were recruited and randomised to SMBP or usual care. This secondary analysis included 91 women randomised to self-monitoring who provided BP readings. Trial instructions were for daily SMBP.

**Main outcome measures:**

Adherence was calculated as proportion of days on which SMBP readings were taken. Proportion of weeks in which at least 4 and at least 2 SMBP readings were taken was also calculated. Mean differences between clinic and SMBP measurements were calculated.

**Results:**

Self-monitored BP data were available for 49 women with chronic hypertension and 42 women with gestational hypertension. Median percentage of days with SMBP readings was 77% (IQR 51, 89) in the chronic hypertension group and 85% (IQR 52, 95) in the gestational hypertension group. Adherence did not vary by different socio-demographic groups. Mean difference (95% CI) between clinic and SMBP for systolic BP was 0.99 mmHg (−1.44, 3.41; chronic hypertension) and 3.76 mmHg (0.75, 6.78; gestational hypertension) and for diastolic BP was 3.03 mmHg (0.93, 5.12; chronic hypertension) and 3.27 mmHg (0.56, 5.98; gestational hypertension).

**Conclusions:**

Adherence to self-monitoring was good and differences between SMBP and clinic readings were small. These findings offer reassurance about the use of self-monitoring at a time when it is being increasingly implemented in maternity settings.

## Highlights

• Chronic hypertension and gestational hypertension groups had good adherence to self monitoring of blood pressure (SMBP).
• There was no evidence of adherence varying in different socio-demographic groups.
• The differences between SMBP and clinic BP readings were small.

## 1 Introduction

Hypertension affects around 10% of pregnancies and is associated with adverse maternal and fetal outcomes [1], [2], [3]. High blood pressure (BP) can develop between antenatal appointments and may be asymptomatic until an advanced stage, and BP self-monitoring might allow for the earlier detection and improved management of pregnancy hypertension [3]. Other possible benefits of self-monitoring include a potential reduction in the need for clinic visits, reducing healthcare expense as well as being more convenient for women [4], [5]. Studies in the non-pregnant population have shown that self-monitoring of blood pressure (SMBP) combined with self-titration of medication can be effective and lead to better blood pressure control than relying on clinic visits [6], [7], [8]. The recent COVID-19 pandemic has also highlighted the benefit and sometimes necessity of people being able to self-monitor in order to reduce their attendances at health services, and has led to more widespread use of SMBP in pregnancy, particularly in women with hypertension [9].

There is limited information on feasibility, adherence and outcomes of SMBP in the pregnant population which perhaps leads to reluctance of clinicians to trust women to monitor sufficiently or effectively [5], [10]. In addition, there is insufficient evidence to date on whether there should be differences between home and clinic thresholds used for diagnosis and management of pregnancy hypertension, as is the case for the general adult population [11], [12].

OPTIMUM-BP was a randomised controlled trial which investigated the feasibility and acceptability of BP self-monitoring for the antenatal control of blood pressure in pregnant women with hypertension [13]. This is a secondary analysis of the results, with the aim of looking firstly at adherence and monitoring behaviour of women undertaking SMBP, and secondly at comparisons between SMBP and clinic readings.

## 2 Methods

### 2.1 Study design

OPTIMUM-BP was an unmasked randomised controlled clinical trial including pregnant women with chronic or gestational hypertension. Full details of study methodology are available in the main results paper [13]. Women from four maternity units in England were recruited and randomised to blood pressure self-monitoring or usual care between December 2015 and December 2017. BP was measured and data collected on demographics, medical and obstetric history, antihypertensive therapy and socio-economic status (including Index of Multiple Deprivation [14]).

Women allocated to usual care had their BP monitored by their local midwifery and obstetric teams. The average of up to three recorded clinic readings on any single occasion was taken as the clinic BP for that episode. Women randomised to the intervention were additionally asked to measure their BP daily, at approximately the same time, using an appropriately sized cuff with a validated automated BP monitor (Microlife WatchBP Home, validated in pregnancy and preeclampsia) [15]. Participants were asked to take two readings at least one minute apart, and to record the second in their study diary. In the gestational hypertension group, they also had the option to submit the reading via text or study app on their mobile phone. The digitally reported readings were automatically transmitted to a secure server, which provided immediate automated responses [13]. Women were provided with guidance about normal and out of range readings and an algorithm based on UK NICE Hypertension in Pregnancy Guidelines [16].

All women were also asked to attend up to three antenatal study visits at 20, 28 and 34 weeks’ gestation, and one six week postnatal face to face or telephone study visit. BP measurement was taken by the study team at these visits using the same model of validated monitor [15].

The study protocol and materials were approved by a UK Research Ethics Committee (15/EM/0490) and research and development approvals gained. All study participants provided written, informed consent. Trial registration: ISRCTN16018898.

### 2.2 Inclusion in analysis

For this secondary analysis, 91 women who were randomised to self-monitoring and provided SMBP readings (diary, app or downloaded BP monitor data) were included. BP measurements with systolic BP (SBP) < 70 or > 260 mmHg or with diastolic BP (DBP) < 40 or > 260 mmHg were discarded as erroneous [17]. The chronic hypertension group and gestational hypertension group were analysed separately due to the differences in protocol.

### 2.3 Statistical analysis

#### 2.3.1 Adherence

Adherence was calculated as the percentage of days enrolled in the study on which participants provided SMBP data. Further adherence variables were calculated as proportion of weeks that women provided at least 4 or at least 2 SMBP readings. Median adherence and quartiles were calculated. Differences in adherence (using the percentage of days SMBP data provided adherence variable) by demographic and pregnancy specific factors were then assessed using nonparametric tests of the equality of medians.

For subjects with downloaded monitor readings, each diary/app reading was compared with monitor readings, to calculate the proportion that were matched to one of the monitor readings and the proportion within 5 mmHg. The proportion of differences that impacted upon the ‘action colour’ (ie would have affected management per the trial alert algorithm, Table S1) was also calculated.

#### 2.3.2 Clinic-SMBP differences

The mean difference with 95% confidence intervals was calculated between each clinic visit and the average SMBP readings for the seven days prior to that clinic. If the previous clinic visit was within the preceding 7 days, only the SMBP readings following the last clinic were included, to account for the fact that there may have been medication changes at the previous clinic. Where more than one source of blood pressure reading for a given woman was available, downloaded monitor readings were used in preference to app readings, which were used in preference to diary readings. As participants were told to ignore the first SMBP reading and to record the second, any first readings on a given occasion were excluded unless that was the only reading of the day; all other readings were included.

Two sensitivity analyses were done. The first used only SMBP readings taken on the same day as the clinic readings. The second used only the second SMBP reading, excluding any subsequent readings taken.

Mean (95% confidence intervals) SMBP and clinic BP readings were plotted by gestation for participants who had both SMBP and clinic BP data in the same week of pregnancy. Bland Altman plots were constructed to compare SMBP and clinic readings by BP level [18], [19].

In the chronic hypertension group, mean clinic readings were also compared to mean study visit readings where blood pressure had been measured systematically three times and a mean taken of all three readings (at 28 and 34 weeks’ gestation; insufficient numbers to look at the 20 week study visit). This was not done for the gestational hypertension group as the majority were recruited after 34 weeks (after the date of the last potential study visit).

All analyses were performed using STATA 16 (Stata-Corp, College Station, Texas, USA).

## 3 Results

### 3.1 Inclusion

**Fig. 1:**
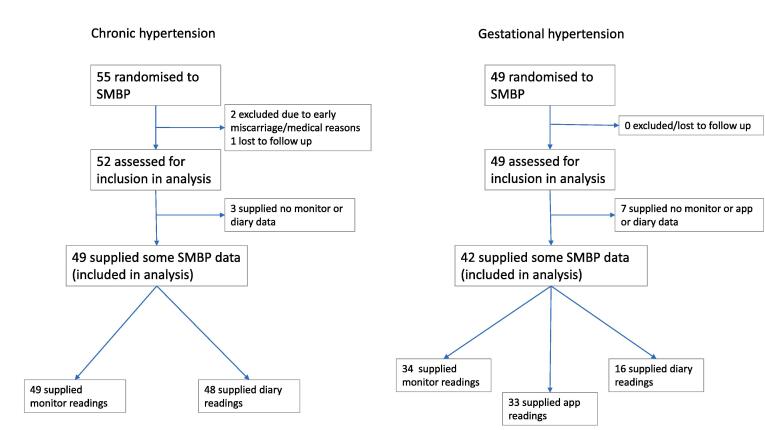
Flow diagram of inclusion in analysis.

Of the 154 women randomised in the OPTIMUM-BP study, 55 women with chronic hypertension and 49 women with gestational hypertension were randomised to self-monitoring. Of the 55 women with chronic hypertension, 2 were excluded due to early miscarriage or medical reasons, 1 lost to follow up, and 3 did not supply any self-monitoring data, leaving 49 (89%) women with chronic hypertension who provided SMBP data and were included in this analysis. Of the 49 women with gestational hypertension randomised to self-monitoring, 7 did not provide any SMBP data, leaving 42 (86%) women included in this analysis (Fig. 1). Demographic and clinical characteristics at enrolment of participants are shown in Table 1.

**Table 1:** Demographic and clinical characteristics at enrolment of pregnant women with hypertension who self-monitored blood pressure in the OPTIMUM-BP feasibility trial.

	Chronic Hypertension (n = 49)	Gestational Hypertension (n = 42)
Age (years), mean (SD)	36.0 (5.4)	32.9 (5.8)
Gestation at recruitment, median (IQR)	16.6 (13.0, 20.3)	34.6 (31.7, 36.0)
BMI (kg/m^2^), mean (SD)	31.1 (6.4)	30.1 (7.2)
*Ethnicity, n (%)*		
White	25 (51)	31 (74)
Black	18 (37)	10 (24)
Asian	6 (12)	1 (2)
Most deprived deprivation quintile*, n(%)	17 (35)	13 (31)
Professional/Higher education, n(%)	34 (69)	32 (76)
Never smokers, n(%)	35 (71)	35 (83)
*Other self-reported medical history, n(%)*		
Diabetes type 2	2 (4)	1 (2)
Chronic kidney disease	1 (2)	0 (0)
Nulliparity, n(%)	16 (33)	19 (45)
Diagnosis of preeclampsia or gestational hypertension in previous pregnancy, n(%)	17 (35)	13 (31)
Proportion prescribed antihypertensive medication at enrolment, n(%)	36 (73)	26 (62)
Previous SMBP in this pregnancy at least once, n(%)	22 (45)	11 (26)

Abbreviations: SMBP: self-monitoring of blood pressure; SD: standard deviation; IQR: Interquartile range.

*Deprivation measured as Index of Multiple Deprivation score using Office of National Statistics for England data.

### 3.2 Adherence

In women with chronic hypertension the median percentage of days that SMBP readings were supplied was 77% (IQR 51–89). A median of 81% (IQR 71–96) of weeks included four or more SMBP readings, and a median of 94% (IQR 78–100) of weeks included two or more SMBP readings (Table 2). In the gestational hypertension group these figures were 85% (IQR 52–95), 80% (IQR 50–100) and 91% (IQR 80–100) respectively. Median interval between SMBP readings was 1 day (IQR 1–1) in both chronic and gestational hypertension groups compared to median interval between clinic readings of 8 days (IQR 7–13) in the chronic hypertension group and 4.5 days (IQR 3.5–7) in the gestational hypertension group (Table S2).

**Table 2:** Percentage of time that participants submitted SMBP readings (monitor or diary or app) between randomisation and delivery, and comparisons between monitor and diary/app readings.

	Chronic Hypertension (n = 49)	Gestational Hypertension (n = 42)
**Adherence with SMBP**		
Percent of days with SMBP readings, Median (IQR)	77 (51, 89)	85 (52, 95)
Percent of weeks with 4 + SMBP readings, Median (IQR)	81 (71 ,96)	80 (50, 100)
Percent of weeks with 2 + SMBP readings, Median (IQR)	94 (78, 100)	91 (80, 100)
Monitor vs diary/app readings*		
Diary/app reading matches a monitor reading exactly	88%	87%
Diary/app readings matches a monitor reading within 5 mmHg	95%	98%
Diary/app reading matches *second* monitor reading exactly	42%	39%
Diary/app readings matches *second* monitor reading within 5 mmHg	66%	58%

* In chronic hypertension group based on 3456 observations from 47 participants; in gestational hypertension group based on 419 observations from 27 participants

When looking at possible factors associated with adherence, there was no evidence of difference by age, body mass index, ethnicity, education or smoking in the chronic or gestational hypertension group (Table 3). When considering pregnancy related factors, there was no evidence of difference in adherence by parity, previous hypertensive disorder of pregnancy, medication at enrolment, or previous self-monitoring in this pregnancy in either group. In the gestational hypertension group, there was some evidence of increased adherence in the group recruited at later gestation.

**Table 3:** Median adherence (percentage of days between randomisation and delivery that women used SMBP) by demographic and pregnancy specific factors.

Variable	Chronic Hypertension	p-value	Gestational Hypertension	p-value
Demographic factors				
Age				
20–34	82 (50, 94)	0.07	85 (70, 95)	0.9
35–44	71 (51, 89)		81 (33, 95)	
45+	71 (37, 76)			
BMI				
<30	75 (50, 88)	0.5	86 (73, 95)	0.8
30+	81 (51, 94)		71 (48, 94)	
Ethnicity				
White	83 (66, 94)	0.1	86 (48, 95)	0.2
Black	62 (23, 94)		69 (52, 91)	
Asian	76 (68, 79)		96 (96, 96)	
Education				
No formal qualifications	12 (5, 19)	0.3	10 (10, 10)	0.6
School qualifications	67 (36, 95)		88 (77, 95)	
Professional/Higher qualifications	79 (67, 89)		85 (50, 95)	
Smoking				
Current smoker	88 (64, 93)	0.6	10 (10, 10)	0.3
Current non-smoker	77 (50, 91)		86 (66, 95)	
Pregnancy specific factors				
Gestation at recruitment				
<15 weeks (CHTN)	78 (66, 94)	0.9	–	
≥15 weeks (CHTN)	76 (50, 89)		–	
<32 weeks (GH)	–		48 (31, 71)	0.005
≥32 weeks (GH)	–		90 (77, 96)	
Parity				
0	77 (67, 83)	0.6	85 (52, 96)	0.8
≥1	81 (41, 94)		86 (48, 95)	

Previous HDP				
No	75 (50, 89)	0.8	79 (48, 92)	0.4
Yes	68 (41, 87)		91 (71, 94)	
Medicated at enrolment				
No	77 (32, 83)	0.8	81 (44, 95)	0.7
Yes	78 (54, 94)		86 (66, 95)	
Previous SMBP in this pregnancy				
No	78 (41, 89)	0.6	86 (52, 96)	0.7
Yes	76 (66, 89)		85 (47, 95)	

The median number of readings per day was 2 (IQR 2–2, range 0–17) in the chronic hypertension group and was 2 (IQR 2–4, range 0–25) in the gestational hypertension group.

When readings retrieved from monitor downloads were compared to those submitted in the diary on the same day, 3032/3456 (88%) of diary readings matched one of the monitor readings exactly, and 3282/3456 (95%) matched one of the monitor readings within 5 mmHg in the chronic hypertension group (Table 2). In the gestational hypertension group 365/419 (87%) of app/diary readings matched one of the monitor readings exactly, and 409/419 (98%) matched one of the monitor readings within 5 mmHg. When comparing the diary/app recorded reading with the second monitor reading specifically, 2270/3456 (66%) of the readings in the chronic hypertension group matched within 5 mmHg and 242/419 (58%) of the readings in the gestational hypertension group matched within 5 mmHg. In 316/3456 (9%) of the chronic hypertension diary readings and 34/419 (8%) of the gestational hypertension app/diary readings, these differences were associated with the monitor readings having a higher ‘action colour,’ ie would have affected automated prompts to action for the women compared to the diary/app reading (as shown in the Blood Pressure Threshold Algorithm, Table S1).

### 3.3 Clinic vs SMBP readings

Mean difference between clinic and SMBP readings was 0.99 mmHg (95% CI −1.44, 3.41) for SBP and 3.03 mmHg (0.93, 5.12) for DBP in the chronic hypertension group. In the gestational hypertension group the mean difference in SBP was 3.76 mmHg (0.75, 6.78) and in DBP was 3.27 mmHg (0.65, 5.98). Sensitivity analyses looking at including only the second reading of the day from monitor readings, and only including readings done on the same day as clinic, did not substantively change the results (Table S3).

The median of the differences between the maximum and minimum readings in clinic SBP taken on the same day was 10 mmHg (IQR 5.5, 15) and in DBP was 5 mmHg (IQR 2, 10).

**Fig. 2:**
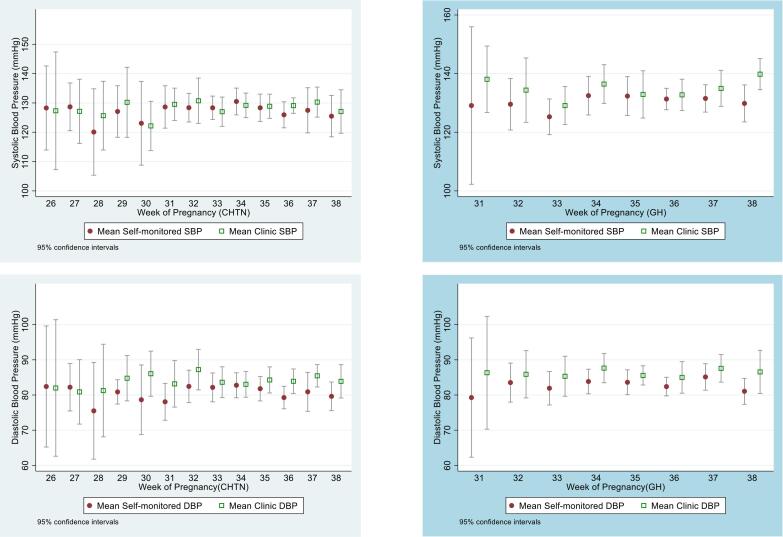
Clinic and SMBP readings throughout pregnancy in chronic hypertension (left) and gestational hypertension (right) groups.

**Fig. 3:**
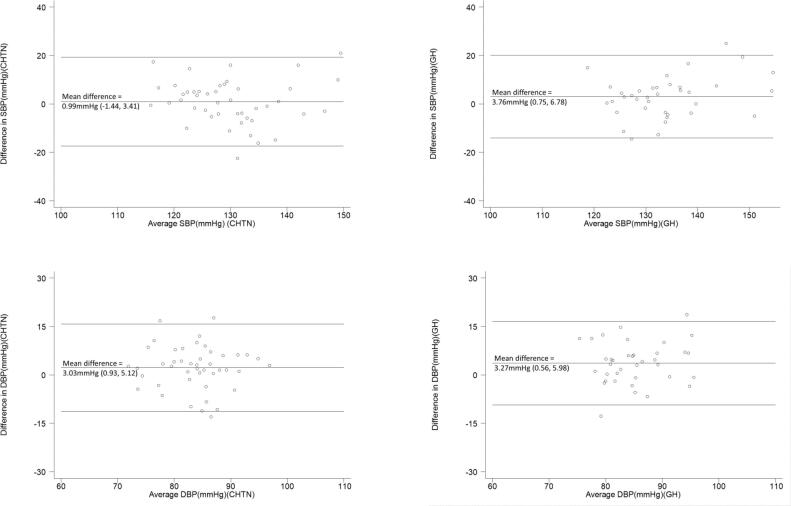
Bland Altman plots showing difference between clinic and SMBP readings (in the 7 days preceding clinic) against mean blood pressure readings Middle line represents mean difference between clinic and SMBP blood pressure readings, outer lines represent the mean difference +/- 1.96 standard deviation (limits of agreement) Participants included where SMBP and clinic readings available from the same week (178 observations for 45 participants in chronic hypertension group; 103 observations from 37 participants in the gestational hypertension group 95% CIs adjusted for clustering by participant.

SMBP and clinic readings plotted across different gestations were not suggestive of evidence of increasing differences between SMBP and clinic readings as gestation increased (Fig. 2). Considering clinic-SMBP differences by average blood pressure, Bland Altman plots showed some suggestion that at higher average BP readings there was more difference between SMBP and clinic (as evidenced by slightly more data points in the top right quarter of the plot, Fig. 3).

Results for differences between clinic and study visit blood pressures showed no evidence of difference (Table S4, Fig. S3).

## 4 Discussion

### 4.1 Main findings

This analysis of adherence to self-monitoring of blood pressure in pregnancy has shown that both chronic hypertension and gestational hypertension groups had good adherence with a low level of inaccurate reporting. There was some suggestion that the gestational hypertension group were more likely to do daily readings and take more readings each day, perhaps because they were monitoring for shorter durations and that hypertension diagnosis and monitoring was a new experience for them. An extension of this was the especially high levels of adherence in the subgroup of women with gestational hypertension who joined the study after 32 weeks’ gestation, who would have only been monitoring for around five to six weeks. It was of interest that other than this subgroup, there was no evidence of differences in adherence by other demographic or pregnancy factors. However, around one in ten monitor readings were higher than those submitted, though these instances were observed infrequently across many women, rather than being clustered in certain individuals. This may have been due to reasons such as women additionally checking their BP prior to taking medication or following certain activities, over and above what was required within the self-monitoring instructions. Overall, differences between SMBP and clinic BP readings were small, with no variation by gestation, although there was some suggestion of greater differences at higher blood pressures as might be expected. These systolic/diastolic home-clinic BP differences (around 3/3 mmHg) were smaller than differences within same day clinic readings (around 10/5 mmHg), and so are unlikely to substantially impact on clinical management, within the wider interpretation of all BP readings. In the absence of data to suggest clinically significant differences between clinical and SMBP readings, this would support using the same thresholds for acting on SMBP readings as on clinic readings.

### 4.2 Strengths and limitations

A strength of this study is the detailed information recorded on BP self-monitoring practices in women with both chronic and gestational hypertension during pregnancy, with the use of a validated blood pressure monitor for pregnancy. Four centres participated (both teaching and general hospitals) with a multi-ethnic population albeit of higher than average education. This gives some reassurance for generalisability although the population was relatively small reflecting that it comprised a feasibility study. This may have limited the power to detect differences between groups, particularly subgroups.

### 4.3 Previous literature

One other antenatal randomised controlled trial of SMBP during pregnancy has been done, which included low risk women rather than women with hypertension [20]. They undertook SMBP on a weekly basis, and results showed a mean number of missed weeks of BP measurements of 0.8 (SD 1.2). No comparisons between monitors and reported readings, or between SMBP and clinic readings were reported. In comparison, women in the current study managed at least two readings per week a median of 90% of the weeks that they were monitoring, although adherence dropped off compared to the daily readings requested.

A systematic review and individual patient data metanalysis assessing differences between clinic and SMBP readings in pregnancy found similarly small mean differences overall (1–2 mmHg)[11]. The subgroup analysis of women with hypertension in pregnancy showed bigger differences than seen here between clinic and SMBP readings, although heterogeneity was high and only 2 of the 7 studies used blood pressure monitors validated for use in pregnancy. A subsequent prospective observational study on a healthy normotensive pregnant population in Denmark, compared self-monitoring done at three discrete times in pregnancy for three days at a time [21]. Mean differences between clinic and SMBP measurements were 11 mmHg in SBP and 9 mmHg in DBP (i.e. higher in clinic than SMBP). The Bland–Altman plots showed increasing difference at higher BPs, similar to the analyses presented here, as well as in the wider adult population [22]. The bigger differences between SMBP and clinic measurements compared to the current study may reflect the shorter duration of monitoring that means women do not become so used to blood pressure monitoring. Another more recent cohort study comparing SMBP and clinic readings in women with hypertension in pregnancy in a UK maternity unit has also been reported [23]. Clinic readings were compared with a single home reading taken closest to the clinic measurement. Mean differences of 7.3 mmHg in SBP and 4.3 mmHg in DBP were seen (i.e. higher in clinic than SMBP), with slightly greater differences at earlier gestations and no clear variation by average BP viewed in Bland-Altman plots [23]. It is not clear why the differences between clinic and SMBP readings were lower in the analyses presented in this paper than in other studies looking at clinic-SMBP differences in pregnant populations with hypertension. Sample sizes were not large in this or the other previous studies, which is known to increase heterogeneity between studies, and larger studies evaluating differences would be beneficial. Another possible contributor is that the analysis in this paper used downloaded monitor readings where these were available, in preference to readings documented in participants’ diaries or apps, whereas most of the studies compared above used self-reported SMBP readings when comparing to clinic readings, that were not independently checked.

### 4.4 Clinical and research implications

The findings of this analysis offer reassurance regarding adherence to self-monitoring in a pregnant population with hypertension, a finding not limited to particular subgroups of women. Generalisability of the results may have been limited by the fact that women consenting to join the study may be more motivated or accepting of the idea of self-monitoring. Some women may choose not to undertake SMBP when offered, or may discontinue once started, typically for reasons such as perceived lack of time or concern that the monitoring was increasing stress, but the majority are willing and motivated to monitor. Further work is needed to optimise equity of SMBP uptake across diverse groups of women, and to empower women to persist, through better understanding reasons for discontinuation. The differences seen between clinic and SMBP readings were less than has been reported in other studies of women with hypertension in pregnancy. Larger studies are needed to elucidate this question. The recent implementation of self-monitoring due to the COVID pandemic should also be seen as an opportunity to further evaluate self-monitoring practices in women with hypertension in pregnancy.

## 5 Conclusion

Adherence to self-monitoring in this population was good and differences between SMBP and clinic readings were small. These findings offer some reassurance about the use of self-monitoring at a time when it is being increasingly implemented in maternity settings.

## Funding

This article represents independent research. RM and LC are supported by Research Professorships from the National Institute of Health Research (NIHR-RP-R2-12-015 and RP-2014-05-019 respectively). RM holds a current National Institute for Health Research Programme Grant for Applied Research (RP-PG-0614-20005), on which KM is a co-applicant. RM, LP and KM have received funding from the National Institute for Health Research Applied Research Collaboration Oxford and Thames Valley. LB is supported by an NIHR Academic Clinical Fellowship in Primary Care. The views expressed in this publication are those of the authors and not necessarily those of the NHS, the NIHR or the Department of Health.

## Declaration of Competing Interest

The authors declare that they have no known competing financial interests or personal relationships that could have appeared to influence the work reported in this paper.
