# Supplementary material for: Adherence with blood pressure self-monitoring in women with pregnancy hypertension, and comparisons to clinic readings: A secondary analysis of OPTIMUM-BP
Source: Pregnancy Hypertens. 2021 Aug;25:68–74. doi: 10.1016/j.preghy.2021.05.016 (PMC8372528; doi:10.1016/j.preghy.2021.05.016)
Supplement: Supplementary data 1 [file mmc1.docx]

**Supplementary Appendix**

**Table S1: BP Threshold Algorithm (action colours)**


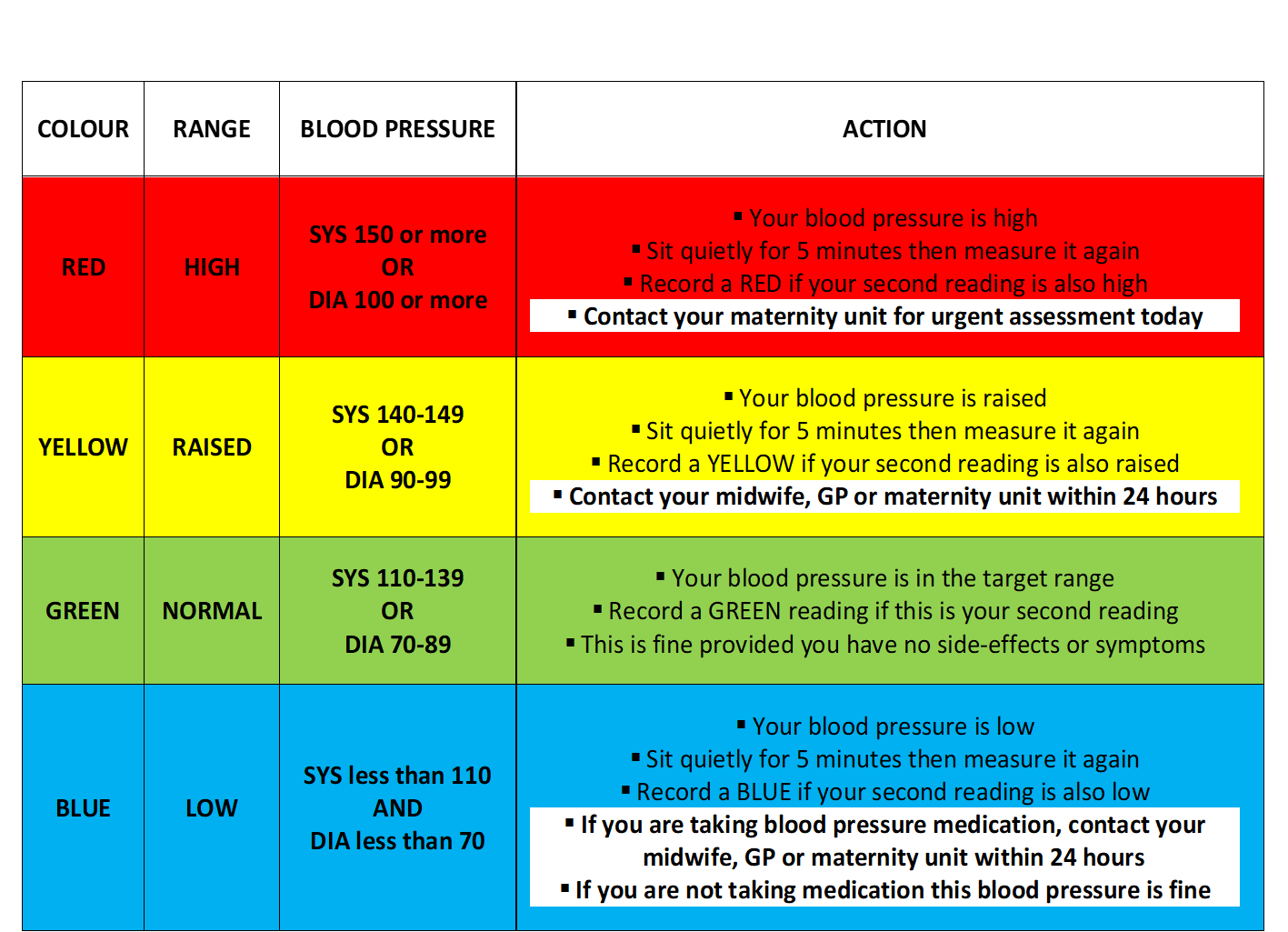


**Figure S1: Histogram of percentage of time that participants submitted SMBP readings (monitor or diary or app) for chronic hypertension and gestational hypertension groups**

**Table S2: Average interval between SMBP and clinic blood pressure readings**

|  | **Chronic Hypertension** | | **Gestational Hypertension** | |
| --- | --- | --- | --- | --- |
| Interval between readings (days) | SMBP | Clinic | SMBP | Clinic |
| Median (IQR) | 1 (1,1) | 8 (7,13) | 1 (1, 1) | 4.5 (3.5, 7) |
| Mean (SD) | 2.3 (3.8) | 14.7 (8.6) | 1.3 (0.5) | 6.2 (3.8) |

**Figure S2: Distribution of difference in blood pressure between the clinic and SMBP mean readings (in the 7 days preceding clinic)**

*Red dashed line represents mean difference; positive numbers represent higher clinic than SMBP readings*

**Table S3: Mean difference between clinic and average SMBP readings (sensitivity analyses looking at a) only SMBP readings from same day as clinic and b) only SMBP readings that were the 2^nd^ reading taken each day)**

|  | Chronic Hypertension | Gestational Hypertension |
| --- | --- | --- |
| SMBP reading from same day as clinic |  |  |
| Number of participants included in comparison* | N=41 | N=34 |
| Number of observations included | 143 | 108 |
| Mean (95% CI^‡^) difference in SBP (mmHg) | -0.47 (-2.97, 2.04) | 2.65 (-0.52, 5.35) |
| Mean (95% CI^‡^) difference in DBP (mmHg) | 3.02 (0.51, 5.52) | 2.28 (0.25, 4.31) |
| Average SMBP including only 2^nd^ reading of each day † |  |  |
| Number of participants included in comparison** | N=45 | N=29 |
| Number of observations included | 163 | 80 |
| Mean (95% CI^‡^) difference in SBP (mmHg) | 0.45 (-1.80, 2.69) | 4.47 (0.86, 8.07) |
| Mean (95% CI^‡^) difference in DBP (mmHg) | 2.72 (0.75, 4.70) | 4.37 (1.23, 7.51) |

* Included where SMBP and clinic readings available from the same day

† Average of the 2^nd^ SMBP readings of each day, from the 7 days prior to clinic

‡ CIs adjusted for clustering by participant

** Included where SMBP and clinic readings available from the same week

**Table S4: Mean difference between clinic and study BP readings**

|  | **Chronic Hypertension (n=35)** | |
| --- | --- | --- |
|  | **SBP** | **DBP** |
| Mean (95% CI) difference (mmHg) | -1.84 (-6.18, 2.51) | -2.01 (-4.57, 0.55) |
| Median (IQR) difference (mmHg) | -0.67 (-4.67, 3.00) | -1.33 (-3.67, 1.00) |

**Figure S3: Study and Clinic blood pressure readings at different gestations (Chronic hypertension group, n=15 for 28 week visit, n=20 for 34 week visit)**

**Figure S4: Mean number of monitor readings per day**
